# Target gene selectivity of hypoxia-inducible factor-α in renal cancer cells is conveyed by post-DNA-binding mechanisms

**DOI:** 10.1038/sj.bjc.6603675

**Published:** 2007-03-27

**Authors:** K W Lau, Y-M Tian, R R Raval, P J Ratcliffe, C W Pugh

**Affiliations:** 1 The Henry Wellcome Building for Molecular Physiology, Oxford, UK

**Keywords:** hypoxia-inducible factor, transcription, von Hippel–Lindau, renal carcinoma, chromatin immunoprecipitation

## Abstract

Inactivation of the von Hippel–Lindau tumour suppressor in renal cell carcinoma (RCC) leads to failure of proteolytic regulation of the α subunits of hypoxia-inducible factor (HIF), constitutive upregulation of the HIF complex, and overexpression of HIF target genes. However, recent studies have indicated that in this setting, upregulation of the closely related HIF-*α* isoforms, HIF-1*α* and HIF-2*α*, have contrasting effects on tumour growth, and activate distinct sets of target genes. To pursue these findings, we sought to elucidate the mechanisms underlying target gene selectivity for HIF-1*α* and HIF-2*α*. Using chromatin immunoprecipitation to probe binding to hypoxia response elements *in vivo*, and expression of chimaeric molecules bearing reciprocal domain exchanges between HIF-1*α* and HIF-2*α* molecules, we show that selective activation of HIF-*α* target gene expression is not dependent on selective DNA-binding at the target locus, but depends on non-equivalent C-terminal portions of these molecules. Our data indicate that post-DNA binding mechanisms that are dissimilar for HIF-1*α* and HIF-2*α* determine target gene selectivity in RCC cells.

Hypoxia-inducible factor is a family of *α*/*β* heterodimeric DNA-binding complexes that act as transcriptional master regulators of the response to low oxygen tensions (Wang *et al*, 1995). Hypoxia-inducible factor activity is upregulated in cancer by a variety of processes including microenvironmental hypoxia, tumour suppressor gene action, growth factor activity and cellular deficiencies of ascorbate and iron. Increased expression of the regulatory HIF-*α* subunits has been associated with tumour aggression and poor prognosis, leading to interest in defining ways of downregulating the pathway as a potential therapeutic strategy in cancer (for review see Harris, 2002; Semenza, 2003).

Hypoxia-inducible factor-*α* chains are encoded by three independent loci. All three gene products dimerise with constitutively expressed HIF-*β* chains, also known as aryl hydrocarbon receptor nuclear translocators. This heterodimer binds to DNA and recruits the p300/CBP coactivator proteins to form an active transcriptional complex. Oxygen-dependent proteolytic regulation of HIF-*α* chain stability is achieved via a prolyl hydroxylase (PHD 1-3)/von Hippel–Lindau (VHL) tumour suppressor protein ubiquitin ligase/proteasome pathway, while coactivator recruitment is controlled by the HIF asparaginyl hydroxylase, factor inhibiting HIF (FIH) that catalyses hydroxylation of an asparagine residue in the carboxy-terminal (C-terminal) activation domain, blocking association with p300/CBP in the presence of oxygen (for review see Hirota and Semenza, 2005; Schofield and Ratcliffe, 2005).

Hypoxia inducible factor-1*α* and HIF-2*α* are the best studied HIF-*α* isoforms. They have a highly conserved domain architecture, including sites of prolyl and asparaginyl hydroxylation, and strongly promote transcription from similar hypoxia response elements (HREs). However, there is increasing evidence for the functional non-equivalence of HIF-1*α* and HIF-2*α*, and in cancer, several recent studies have indicated that, at least in certain settings, they have contrasting effects on tumour growth (Maranchie *et al*, 2002; Kondo *et al*, 2002, 2003; Zimmer *et al*, 2004; Covello *et al*, 2005; Raval *et al*, 2005). In VHL-defective renal carcinoma cells (RCC), inactivation of HIF-*α* proteolysis upregulates both HIF-1*α* and HIF-2*α* with global induction of HIF target gene expression (Maxwell *et al*, 1999), stimulating investigations of the role of the HIF pathway in tumour development (Maranchie *et al*, 2002; Kondo *et al*, 2002, 2003; Zimmer *et al*, 2004; Raval *et al*, 2005). These studies have shown that suppression of HIF-2*α* retards and overexpression of HIF-2*α* enhances the growth of experimental tumours derived from RCC cells. In contrast, overexpression of HIF-1*α* was found to retard the growth of similar RCC-derived experimental tumours. Interestingly, clinical RCC shows an unusual bias to greater HIF-2*α* rather than HIF-1*α* expression (Krieg *et al*, 2000; Turner *et al*, 2002), and in kidneys from patients with VHL disease, greater HIF-2*α* expression is associated with more advanced lesions (Mandriota *et al*, 2002). Taken together, these findings strongly suggest that HIF-2*α* has pro-tumorigenic actions in RCC that are isoform specific, and not shared by HIF-1*α*. In keeping with this, HIF-1*α* and HIF-2*α* show clear transcriptional selectivity, with studies to date defining at least two types of isoform-specific responses; certain genes appear exclusively responsive to HIF-1*α* in both RCC and non-RCC cells, whereas others respond to both HIF-1*α* and HIF-2*α* in non-RCC cells, and are dominantly regulated by HIF-2*α* in RCC cells (Hu *et al*, 2003; Sowter *et al*, 2003; Raval *et al*, 2005). Hence, RCC cells display marked HIF-*α* chain selectivity in target gene responses that likely underlie differences in the role of these molecules in promoting tumour growth. Clearly, if the HIF pathway is to be optimally targeted for cancer therapy, it will be important to understand the basis of these differences.

In the current work, we therefore sought to analyse mechanisms underlying selective activation by HIF-1*α* and HIF-2*α*, and demonstrate that selectivity is not a function of differential binding of HIF-1*α* and HIF-2*α* to regulatory HREs, but involves post-DNA-binding mechanisms mediated by more C-terminal regions of the HIF-*α* proteins that are distinct for each HIF-*α* isoform.

## MATERIALS AND METHODS

### Plasmid construction

To generate chimaeric HIF-*α* expression plasmids, site-directed mutagenesis was performed on pcDNA, Hs.HIF-1*α* and pcDNA.Hs.HIF-2*α* (Cockman *et al*, 2000) to introduce novel restriction sites without changing the encoded protein using primers listed in Supplementary Table 1. After confirmation by restriction digestion and DNA sequencing, the sites introduced were used to allow domain exchange by standard recombinant techniques. These chimaeric HIF-*α* sequences were transferred into the retroviral vector pLZRS-IRES-GFP (Jacobs *et al*, 1999). Retroviral production and infection of target cells were conducted as previously described (Raval *et al*, 2005).

A 1.1 kb fragment of the PHD3 first intron (14 328 421 to 14 327 303, antisense strand, NT_026437.10) and a 1 kb fragment of the PHD3 promoter upstream to the translation start site (33 490 609 to 33 489 711, antisense strand, NC_000014.7) were amplified by PCR from human genomic DNA using primers containing the appropriate restriction sites (Sigma Genosys, UK). Primers for the former were 5′-ATggtacc(*Kpn*I)TTGATTGGTAACTTCG 3′- and 5′-ACG aga tct(*Bgl*II) ATA GTT CTA CCA GGC 3′. Primers for the latter were 5′-ACT aga tct(*Bgl*II) TCT TCA TTG TGC TCA TCC CC 3′ and 5′ CGc cat gg(*Nco*I)T CGC CCG CAG AAT CGA GGT CC-3′. PHD3.HRE-SV40-luc was created by cloning the PHD3 first intron fragment into the multiple cloning site of pGL3promoter (Promega, Southampton, Promega, UK). A mutant HRE version of this reporter was created using site-directed mutagenesis with: 5′-CCACCCTCACGCAGCGacatTAGCCCTGTCACTCAGCC-3′ and a complementary oligonucleotide 5′ GGC TGA GTG ACA GGG CTA atg tCG CTG CGT GAG GGT GG 3′ (mutated nucleotides in lower case). Replacement of the SV40 promoter in pGL3promoter with the cloned PHD3 promoter gave rise to PHD3.HRE-PHD3pro-luc. PHD3pro-luc-PHD3.HRE was created by placing the PHD3 first intron fragment into a *Bam*HI site downstream of the poly(A) signal in pGL3promoter using the following primers: 5′-Ggg atc c(*Bam*HI)TC GAG TTG ATT GGT AAC TTC G-3′ and 5′-Ggg atc c(*Bam*HI)AT AGT TCT ACC AGG C-3′. pCMV-*β*-galactosidase (O'Rourke *et al*, 1999), CA9pro.HRE-luc (p506), p173 and p36 (Wykoff *et al*, 2000) have been described previously.

### Cell culture

The VHL competent cell lines used were Caki-1 (spontaneous renal carcinoma; originally from ECACC). Hep 3B (hepatoma containing integrated hepatitis B viral genome; originally from ECACC) and MCF-7 (metastatic mammary adenocarcinoma; originally from ECACC). The VHL defective cell lines 786-O (originally from ATCC), A498 (originally from ECACC) and RCC4 (a gift from CHMC Buys) were all derived from renal clear cell tumours. All cell lines were maintained in Dulbecco's modified Eagles Medium supplemented with 10% fetal calf serum, 2 mM L-glutamine, 50 IU/ml penicillin and 50 *μ*g/ml streptomycin. Stable transfectants of RCC4 containing HRE-linked reporter genes were selected and maintained in 1 *μ*g/ml puromycin. Cells were grown in either normoxia (21% oxygen and 5% carbon dioxide) or hypoxia (1% oxygen and 5% carbon dioxide for 16 h) in an Invivo_2_ hypoxic workstation (Ruskinn Technologies, Bridgend, UK).

### Transient transfections

Two sets of siRNA duplexes targeting HIF-1*α* and HIF-2*α* were used as described previously (Sowter *et al*, 2003; Warnecke *et al*, 2004). The scrambled siRNA duplexes and those targeting *Drosophila melanogaster* HIF-1*α* were used as controls. For reporter assays, cells were plated at 30% density in 12-well plates in antibiotic-free medium on day 0. In siRNA suppression experiments, cells were transfected with siRNA duplexes (20–100 nM) using Oligofectamine reagent (Invitrogen, Paisley, UK) on day 1, cotransfected with siRNA duplexes (20–100 nM), 800 ng luciferase reporter plasmid and 200 ng pCMV-*β*-galactosidase plasmid using Lipofectamine 2000 Reagent (Invitrogen) on day 2, and harvested on day 3. In overexpression experiments, cells were cotransfected with 500 ng HIF-*α* expression plasmid (pcDNA.Hs.HIF-1*α* or pcDNA.Hs.HIF-2*α*), 300 ng HRE-linked luciferase reporter plasmid (PHD3.HRE-PHD3pro-luc, PHD3pro-luc-PHD3.HRE or CA9pro.HRE-luc) and 200 ng pCMV-*β*-galactosidase plasmid using Lipofectamine 2000 reagent on day 1, and harvested on day 2.

Cells were collected in 100 *μ*l 1 × passive lysis buffer (Promega) and assayed for luciferase activity according to the manufacturer's instructions. *β*-Galactosidase assay was performed to normalise for transfection efficiency.

### Immunoblotting

Whole cell extracts from six-well plates were prepared in urea-sodium dodecyl sulphate (SDS) buffer, normalised for total protein content, resolved and transferred as previously described (Raval *et al*, 2005). Primary antibodies were mouse monoclonal antibodies against HIF-1*α* (clone 54, Transduction Laboratories, Oxford, UK), HIF-2*α* (NB-100 132, Novus Biologicals, Littleton, CO, USA), CA9 (M75) (Pastorekova *et al*, 1992), PHD3 (188e) (Appelhoff *et al*, 2004) and *β*-tubulin (clone 2-28-33, Sigma, Gillingham, UK). Horseradish peroxidase-conjugated goat anti-mouse secondary antibody (DAKO, Ely, UK) and ECL Plus system (Amersham Biosciences, Little Chalfont, UK) were used for detection.

### Chromatin immunoprecipitation

Cells were grown in 150-mm dishes to about 80% confluency before being subjected to formaldehyde crosslink, harvest and chromatin immunoprecipitation (chIP) using standard ChIP protocols (Wells and Farnham, 2002; Weinmann and Farnham, 2002). Hep3B cells were treated with desferrioxamine at a final concentration of 100 *μ*M for 16 h before formaldehyde crosslink. The rabbit polyclonal anti-HIF-1*α* (PM14) and anti-HIF-2*α* (PM9) antibodies were used in the IP. PM14 and PM9 were raised by immunising a rabbit with a fusion protein consisting of glutathione- S-transferase fused to amino acids 445–553 of mouse HIF-1*α* or amino acids 357–439 of mouse HIF-2*α*, respectively. Both antisera cross react with the relevant human HIF isoforms. Dilution buffer and rabbit immunoglobulin were used as negative controls. The resulting DNA was analysed by PCR with primers flanking the HREs of PHD3 intron 1 and CA9 promoter (Supplementary Table 1).

For ChIP–Western analysis, urea–SDS lysis buffer was added directly onto the beads used for IP of DNA–protein complexes after the washing steps and boiled at 95°C for 5 min. After centrifugation at 4000 r.p.m. for 5 min, the supernatant was separated by polyacrylamide gel electrophoresis, transferred and blotted with mouse monoclonal antibodies against HIF-1*α* and HIF-2*α*.

## RESULTS

**Figure 1:**
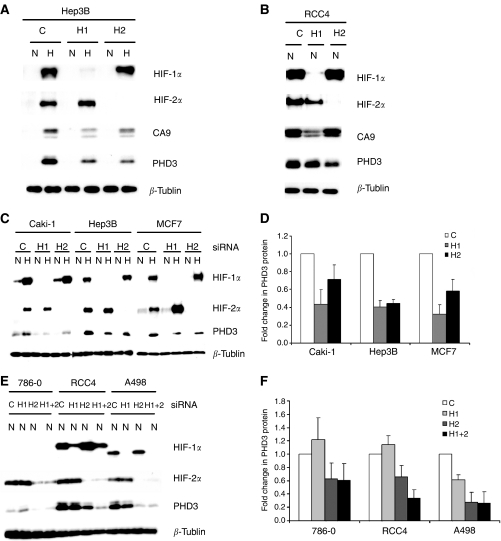
Regulation of PHD3 by HIF-1*α* and HIF-2*α* in renal cell carcinoma (RCC) and non-RCC lines. (**A**) and (**B**) Representative western blots showing HIF-1*α*, HIF-2*α*, CA9, PHD3 and *β*-tubulin protein levels in whole cell lysates of Hep3B and RCC4 cells under conditions of normoxia (N) or hypoxia (H) following siRNA suppression with a control sequence (C) or sequences targeting HIF-1*α* (H1), HIF-2*α* (H2), using siRNA duplexes as described by Warnecke *et al*, 2004. (**C**) and (**E**) Representative western blots showing HIF-1*α*, HIF-2*α*, PHD3 and *β*-tubulin protein levels in whole cell lysates of VHL-competent RCC and non-RCC lines (**C**) and VHL-defective RCC lines (**E**) following siRNA suppression with a control sequence (C) or sequences targeting HIF-1*α* (H1), HIF-2*α* (H2) or both (H1+2), using siRNA duplexes as described by Sowter *et al*, 2003, under conditions of normoxia (N) or hypoxia (H). (**D**) and (**F**) Quantitation of changes in PHD3 protein levels, measured by densitometry, from at least three independent repetitions of this experiment (error bars corresponding to one standard deviation). In the VHL competent cell lines both HIF-1*α* and HIF-2*α* influence PHD3 levels whereas in the VHL-defective cells the effects of HIF-2*α* are predominant.

Previous work with non-RCC cells has indicated that although the large majority of hypoxia inducible genes are responsive to HIF-1*α* (Hu *et al*, 2003; Park *et al*, 2003; Sowter *et al*, 2003; Manalo *et al*, 2005; Wang *et al*, 2005), two distinct patterns of response are observed with some genes being exclusively responsive to HIF-1*α* and some responding to both HIF-1*α* and HIF-2*α*. In RCC cells, this transcriptional selectivity is more marked with the former group of genes remaining exclusively responsive to HIF-1*α* and the latter becoming dominantly, or even exclusively, responsive to HIF-2*α* (Sowter *et al*, 2003; Raval *et al*, 2005). To simplify the analysis of mechanisms underlying these patterns of transcriptional selectivity, we selected two genes, CA9 and PHD3, that manifest particularly strong hypoxia-inducible responses from low basal levels of expression in normoxic non-RCC (VHL competent) cells. CA9 has previously been reported to be induced by HIF-1*α* alone in all cell types (Grabmaier *et al*, 2004; Raval *et al*, 2005), whereas PHD3 had been demonstrated to be regulated by both HIF-1*α* and HIF-2*α* in non-RCC cells, but had not been studied in this way in RCC cells (Aprelikova *et al*, 2004). First, we showed the contrasting behaviour in response to HIF-1*α* and HIF-2*α* siRNA of CA9 and PHD3 in Hep3B and RCC4 cells (Figure 1, panels A and B), using siRNA duplexes as described in Warnecke *et al* (2004). As expected, CA9 expression was dominantly affected by HIF-1*α* siRNA in both cell types. In contrast, PHD3 expression was modulated by both HIF-1*α* and HIF-2*α* siRNA in Hep3B cells, but predominantly by HIF-2*α* siRNA in RCC4 cells. To examine the generality of these effects, we compared the effects of siRNA-mediated suppression of HIF-1*α* or HIF-2*α* on PHD3 expression in a wider selection of RCC and non-RCC cells, using the independent HIF isoform-specific siRNA duplexes described by Sowter *et al* (2003). These experiments confirmed striking induction by hypoxia, and dependence of this response on both HIF-1*α* and HIF-2*α* in non-RCC cells (Figure 1, panels C and D). In contrast, in RCC cells, PHD3 expression was dominantly dependent on HIF-2*α* (Figure 1, panels E and F) whether the cells expressed electrophoretically normal HIF-1*α* (RCC4), HIF-1*α* protein with enhanced electrophoretic mobility (A498) or no immunoreactive HIF-1*α* protein (786-0). The concordance of results produced with independent sets of siRNA duplexes indicates that they are due to specific effects on the targeted isoform. Thus, PHD3 expression conforms to the pattern that has previously been described for HIF target genes such as VEGF and GLUT1 (Raval *et al*, 2005), and indicating that PHD3 would be a suitable gene to study HIF-2*α*-dependent selectivity in RCC cells.

**Figure 2:**
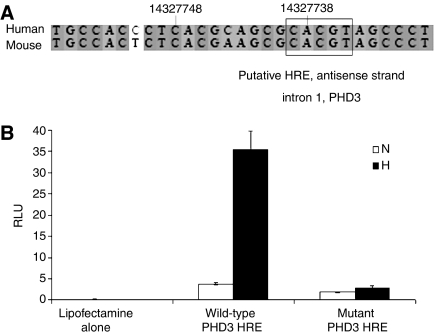
Definition of a hypoxia response element (HRE) within intron 1 of PHD3. (**A**) Alignment of human and mouse sequences showing putative HRE within intron 1 of PHD3 (numbers represent nucleotides of human PHD3 first intron, NT_026437.10). The box outlines a consensus HRE sequence on the antisense strand. (**B**) Luciferase activity (RLU) of whole cell lysates from Hep3B cells transfected with either lipofectamine alone, wild-type PHD3 HRE-linked reporter or mutant PHD3 HRE-linked reporter (ACGTG mutated to AATGT) under conditions of normoxia (N) or hypoxia (H), corrected for transfection efficiency. Values are the mean of three independent repeats and error bars correspond to one standard deviation. The wild-type, but not mutant, HRE sequence confers hypoxia inducible expression of luciferase.

At the time we initiated this work, the location of the hypoxia response element necessary for hypoxic induction of PHD3 expression was unknown. Our *in silico* analysis revealed a consensus HRE in the mid-part of the first intron of the PHD3 gene, which is conserved between mouse and human genomes (Figure 2A). To test the function of this sequence experimentally, we inserted a 1.1 kb genomic fragment containing the putative HRE into pGL3promoter adjacent to a SV40 promoter. Following transient transfection into Hep3B cells, the wild-type sequence, but not a variant containing a mutation in the HRE core motif, conferred hypoxic upregulation of luciferase activity (Figure 2B). The HRE thus defined is in keeping with that identified in recently published data (Pescador *et al*, 2005).

**Figure 3:**
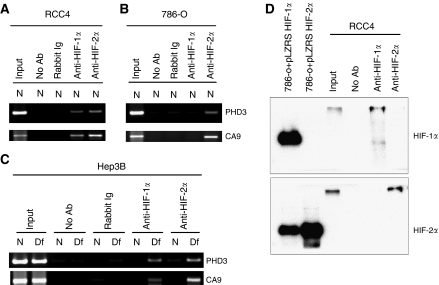
HIF-*α* occupancy of target gene hypoxia response elements (HREs) identified by chromatin immunoprecipitation (ChIP) in RCC and non-RCC lines. (**A**–**C**) ChIP material obtained using rabbit polyclonal antibodies against HIF-1*α* (anti HIF-1*α*) or HIF-2*α* (anti HIF-2*α*) was analysed with PCR primers that amplify either the CA9 or PHD3 HRE in RCC4 (**A**), 786-O (**B**) and Hep3B (**C**) cells under conditions of normoxia (N) or treatment with 100 *μ*M desferrioxamine for 16 h (Df). Controls include reactions using the unfractionated cross-linked DNA-protein complexes (input) and following ChIP without primary antibody (no Ab) and or with normal rabbit immunoglobulin (rabbit Ig). Chromatin immunoprecipitation resulted in selective enrichment of DNA fragments containing CA9 or PHD3 HREs by both HIF-1*α* and HIF-2α suggesting that HIF-*α* transcriptional selectivity is not due to DNA binding selectivity. (**D**) Immunoblots detecting HIF-1*α* (upper panel) or HIF-2*α* (lower panel) of chromatin immunoprecipitated material from RCC4 using antibodies against HIF-1*α*, HIF-2*α* or without primary antibody (no Ab) compared with unfractionated cross-linked DNA-protein complexes (input), confirming the specificity of the anti-HIF-*α* rabbit polyclonal antibodies used. Whole cell lysates of 786-O cells retrovirally overexpressing HIF-1*α* (786-O+pLZRS HIF-1*α*) or HIF-2*α* (786-O+pLZRS HIF-2*α*) were used as controls for the western blot. (Note that the HIF-*α* detected following ChIP migrated more slowly than the control HIF-*α* due to cross-linked DNA.).

To determine whether *in vivo* occupancy of target gene HREs by HIF-*α* isoforms could explain the observed transcriptional selectivity for HIF-1*α* versus HIF-2*α*, we performed chIP studies using rabbit polyclonal antibodies against HIF-1α and HIF-2*α* and PCR primers that amplify either the CA9 or PHD3 HRE. Analysis of the immunoprecipitates from RCC4 cells revealed capture of both CA9 and PHD3 HREs with both HIF-1*α* and HIF-2*α* antibodies (Figure 3A), despite contrasting activation by HIF-1*α* (CA9) or HIF-2*α* (PHD3). Further analysis of immunoprecipitates from 786-O cells revealed enrichment of both HREs by the antibody to HIF-2*α*, but as expected neither HRE was captured in immunoprecipitates using the antibody against HIF-1*α* (Figure 3B), as immunodetectable HIF-1*α* is not present in this cell line. The capture of the CA9 HRE by the antibody to HIF-2*α* in 786-O cells again contrasts with the observed pattern of transcriptional activation, as these cells normally do not express detectable levels of CA9 protein even in the presence of high levels of HIF-2*α* protein. To investigate whether these phenomena were specific to VHL-defective RCC cells, we examined the occupancy of HREs by HIF-*α* isoforms in Hep3B cells, with and without HIF induction following treatment with desferrioxamine. Again, capture of both PHD3 and CA9 HREs was seen with both HIF-1*α* and HIF-2*α* IP (Figure 3C). To exclude the possibility that these results arose from cross-reactivity of the polyclonal anti-HIF-1*α* and anti-HIF-2*α* antisera, we confirmed specificity by analysing the immunoprecipitates from RCC4 cells directly by immunoblotting with independent isoform-specific monoclonal antibodies against HIF-1*α* and HIF-2*α* (Figure 3D). The results indicate that both HIF-*α* isoforms bind *in vivo* to the HREs of target genes predominantly activated by one isoform alone, suggesting that HIF-*α* target gene selectivity in VHL-defective RCC and other cell types is not due to selective DNA binding.

**Figure 4:**
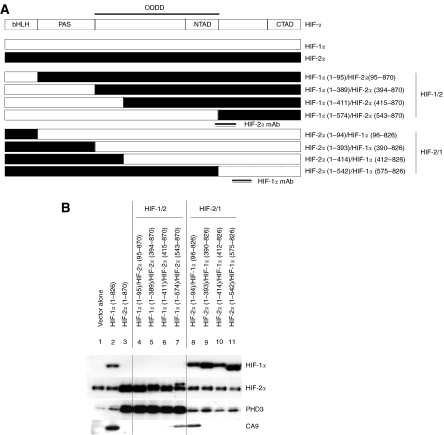
Use of chimaeric HIF-*α* to define HIF-*α* domains determining transcriptional selectivity in 786-O cells. (**A**) Schematic diagram of HIF-*α* chimaeras created in the retroviral expression vector pLZRS-IRES-GFP. Numbers indicate amino acid residues of the N- and C-terminal portions of HIF-1*α* and HIF-2*α* in these chimaeric molecules. Double lines indicate position of epitopes recognised by the anti-HIF-*α* monoclonal antibodies used. (**B**) Representative immunoblots detecting HIF-1*α*, HIF-2*α*, PHD3 and CA9 proteins in whole cell lysates of 786-O cells after retroviral infection with viruses expressing no protein (vector alone), wild-type HIF-1*α* (HIF-1*α* 1-826), wild-type HIF-2*α* (HIF-2*α* 1-870) and the indicated chimaeric HIF-α molecules. Transcriptional selectivity is not determined by HIF-*α* domains involved in DNA binding, but through non-congruent C-terminal portions of these molecules.

To analyse the domains within the HIF-*α* isoform proteins responsible for selectivity, we next analysed the ability of a series of chimaeric HIF-*α* proteins, bearing reciprocal exchanges of major domains between HIF-1*α* and HIF-2*α* (Figure 4A), to activate CA9 and PHD3 in 786-O cells. Retroviral expression of full-length HIF-1*α* but not HIF-2α restored expression of CA9 in 786-O cells. Transfer of the HIF-1α basic-loop–helix domain (amino acids 1–95) implicated in DNA binding into the HIF-2*α* molecule did not enable activation of the CA9 gene. In contrast, replacement of the entire C-terminal HIF-2*α* sequence (amino acids 95–870) with the equivalent HIF-1*α* sequence (amino acids 96–826) was sufficient to convey full activity on the CA9 target gene. Comparison of the effects of the chimaera containing HIF-1*α* amino acids 96–826 with those containing less extensive C-terminal sequences (Figure 4B; lanes 8–11) showed a marked loss of activity as the HIF-1*α* sequence was reduced to amino acids 390–826, thus implicating HIF-1*α* amino acids 96–390 as necessary for expression of CA9. However, consideration of a further series of chimaeras containing progressive extensions of the HIF-1*α* N-terminal sequences (Figure 4B; lanes 4–7) indicated that these sequences alone were not sufficient to restore CA9 expression. Indeed, induction of CA9 expression by a chimaera containing HIF-1*α* amino acids 1–574 but not by a chimaera containing HIF-1*α* amino acids 1–411 indicated that residues between 411 and 574 are also necessary for this response (Figure 4B).

Analysis of PHD3 indicated that in contrast with CA9, but in keeping with results of the siRNA-mediated suppression experiments, PHD3 expression was enhanced by retroviral expression of full-length HIF-2*α* but not by HIF-1*α*. Again, transfer of the HIF-2*α* basic-helix–loop–helix domain (amino acids 1–94) implicated in DNA binding into the HIF-1*α* molecule did not enable activation of the PHD3 gene, whereas replacement of the entire C-terminal HIF-1α sequence with the equivalent HIF-2*α* sequence (amino acids 95–870) was sufficient to convey full activity on the PHD3 target gene. So far the relationship between HIF-1*α* and CA9 and that between HIF-2*α* and PHD3 appeared to be reciprocal. However, further analysis revealed that whereas extension of the N-terminal HIF-2*α* sequence to amino acids 1–542 did not restore PHD3 activation to the chimaera, the HIF-2*α* C-terminal sequences 543–870 conveyed full activity, indicating that these sequences were both necessary and sufficient for HIF-2*α* activation of PHD3 (Figure 4B).

Thus in keeping with the chIP experiments that indicated equivalent DNA binding of HIF-1*α* and HIF-2*α*, transcriptional selectivity was not determined by domains involved in DNA binding but by C-terminal sequences. Interestingly, however, the C-terminal sequences responsible for activation of CA9 or PHD3 were not congruent between the HIF-*α* isoforms, with more extensive sequences being required for the activation of CA9 by HIF-1*α* sequences. These results are in keeping with a role for the HIF-1*α* N-terminal activation domain in the specific activation of the CA9 gene and the HIF-2*α* C-terminal activation domain in activating the PHD3 gene in RCC. However, the chimaeric protein analysis indicates that the HIF-1*α* sequence requirements for CA9 activation are more extensive than the minimal N-TAD, with amino acids 96–390 and 411–574, both being necessary for the response. Note that these experiments do not formally distinguish between activation by C-terminal sequences or repression by the N-terminal sequences, although given existing data mapping activation domains to the C-terminal portions of these molecules (Jiang *et al*, 1996; O'Rourke *et al*, 1999), the former is more probable. These data imply that interactions between the defined domains and one or more proteins are necessary to achieve transcriptional selectivity, presumably resulting from interaction with DNA sequences other than the minimal HRE at the relevant gene locus. This led us to explore the *cis*-acting determinants that might be responsible for transcriptional selectivity at the CA9 and PHD3 loci.

**Figure 5:**
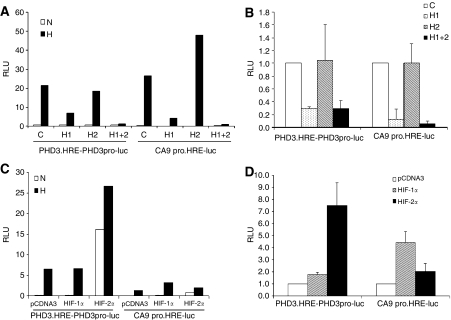
Analysis of *cis*-acting determinants of HIF-*α* transcriptional selectivity through transient transfection of luciferase reporter plasmids containing PHD3 enhancer or CA9 promoter sequences. (**A**) and (**B**) Luciferase activity (RLU) of whole cell lysates of Hep3B (**A**) or fold change in RLU of whole cell lysates in RCC4 (**B**) cells transiently transfected with luciferase reporter plasmids driven by the PHD3 enhancer and promoter (PHD3.HRE-PHD3pro-luc) or the CA9 promoter (CA9 pro.HRE-luc) following siRNA suppression with a control sequence (C) or sequences targeting HIF-1*α* (H1), HIF-2*α* (H2) or both (H1+2) under conditions of normoxia (N) or hypoxia (H), corrected for transfection efficiency. Experiments with RCC4 cells were conducted in normoxia. Expression of both reporter plasmids is reduced when HIF-1*α* is suppressed but not when HIF-2*α* alone is suppressed. (**C**) and (**D**) Luciferase activity (RLU) of whole cell lysates of Hep3B cells (**C**) or fold change in RLU of whole cell lysates in RCC4 (**D**) cells transiently co-transfected with luciferase reporter plasmids driven by the PHD3 enhancer and promoter (PHD3.HRE-PHD3pro-luc) or the CA9 promoter (CA9 pro.HRE-luc) and vectors expressing no protein (pcDNA3) or the indicated HIF-*α* isoforms under conditions of normoxia (N) or hypoxia (H), corrected for transfection efficiency. Experiments with RCC4 cells were conducted in normoxia. Expression of the reporter plasmid driven by the PHD3 enhancer and promoter (PHD3.HRE-PHD3pro-luc) is influenced to a greater extent by HIF-2*α* over-expression than HIF-1*α* over-expression, whereas expression of the reporter plasmid driven by the CA9 promoter is influenced to a greater extent by HIF-1*α* over-expression than HIF-2*α* over-expression. Panels (**A**) and (**C**) are representative histograms. Values in panels (**B**) and (**D**) are mean of three independent experiments, error bars indicate one standard deviation.

We first compared the HIF-*α* isoform dependence of the activity of PHD3.HRE-PHD3pro-luc, containing the PHD3 intronic enhancer as a 1.1 kb fragment and the PHD3 promoter in pGL3promoter with that of a 506 bp fragment encompassing the human CA9 promoter. DNA sequences were transiently transfected into either Hep3B cells or RCC4 cells that had been pre-exposed to siRNAs directed against HIF-1*α* and/or HIF-2*α* so as to achieve specific suppression of one or both HIF-*α* isoforms. Results are summarised in Figure 5 (panels A and B). The DNA sequences behave somewhat differently in that suppression of hypoxic induction by HIF-1α siRNA alone was more effective for CA9 than for PHD3, in both Hep3B cells and RCC4 cells. Surprisingly, however, the most striking finding was that the dominant dependence of HIF-2*α* expression observed for endogenous PHD3 expression in RCC4 cells was not observed for the PHD3 enhancer linked reporter gene expression. Rather, the PHD3 reporter gene expression was most effectively suppressed by siRNA directed against HIF-1*α*.

One potential explanation for the dominant activity of HIF-1*α* siRNA on the 1.1 kb PHD3 intronic enhancer and the 506 bp CA9 promoter is that these elements are simply incapable of responding to HIF-2*α* at all. To test this possibility, we cotranfected these reporters with plasmids expressing either HIF-1*α* or HIF-2*α* in both Hep3B and RCC4 cells. Results shown in Figure 5 (panels C and D) reveal striking activation of the PHD3 intronic enhancer by HIF-2*α* that greatly exceeded that observed with HIF-1*α*. The CA9 promoter also responded to HIF-2*α*, but less well than the PHD3 intronic enhancer.

We did not measure the overall levels of HIF-1*α* and HIF-2*α* achieved in the transiently transfected cell populations. However, it is noteworthy that the levels of HIF-1*α* and HIF-2*α* produced by overexpression had contrasting relative effects on the two reporters, with HIF-1*α* being more active than HIF-2*α* on the CA9 reporter and HIF-2*α* was more active than HIF-1*α* on the PHD3 reporter, suggesting that the results did not simply arise because one, or the other, isoform was more heavily overexpressed.

Taken together, these findings indicate that at least a proportion of the selectivity of the endogenous CA9 gene for HIF-1*α* is conveyed by transiently transfected promoter sequences, but that the selectivity of endogenous PHD3 for HIF-2*α* is not conveyed by a PHD3 promoter/enhancer sequence, at least as configured in PHD3.HRE-PHD3pro-luc, even though this sequence is strikingly responsive to overexpressed HIF-2*α*.

**Figure 6:**
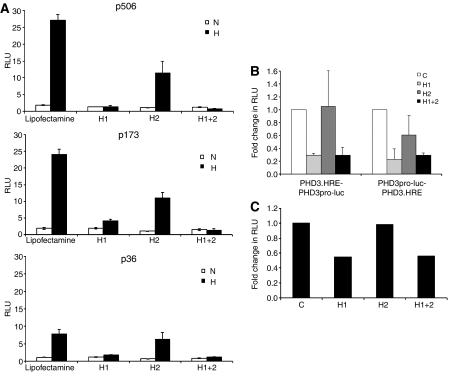
A minimal promoter is sufficient to confer HIF-*α* selectivity in CA9 while additional determinants are required for PHD3. (**A**) Luciferase activity (RLU) of whole cell lysates from Hep3B cells transiently transfected with a series of luciferase reporters containing CA9 promoter sequences containing the indicated number of nucleotides (p506, p173 and p36) following control treatment with lipofectamine alone (lipofectamine) or siRNA suppression with sequences targeting HIF-1*α* (H1), HIF-2*α* (H2) or both (H1+2) under conditions of normoxia (N) or hypoxia (H). (**B**) Fold changes in RLU of whole cell lysates from RCC4 cells transiently transfected with luciferase reporter plasmids driven by the PHD3 enhancer adjacent to the PHD3 promoter (PHD3.HRE-PHD3pro-luc) or driven by the PHD3 enhancer positioned downstream of the PHD3 promoter and luciferase sequence (PHD3pro-luc-PHD3.HRE) following siRNA suppression with sequences targeting HIF-1*α* (H1), HIF-2*α* (H2) or both (H1+2) compared with a control sequence (C) in normoxia. In both panels (**A**) and (**B**), values are the mean of three independent experiments, error bars correspond to one standard deviation. (**C**) Fold change in RLU in a representative RCC4 clone following stable transfection with a luciferase reporter plasmid driven by the PHD3 enhancer positioned downstream of the PHD3 promoter and luciferase sequences PHD3pro-luc-PHD3.HRE following siRNA suppression with sequences targeting HIF-1*α* (H1), HIF-2*α* (H2) or both (H1+2) compared with a control sequence (C) in normoxia. Luciferase activity was normalised with total protein content. Similar results were obtained using an independent stable clone bearing the same plasmid.

To analyse further sequences conveying HIF-1*α* selectivity at the CA9 promoter, we tested the activity of a series of CA9 promoter deletions by transient transfection of Hep3B cells that had been pretreated with siRNAs directed against HIF-1*α* and/or HIF-2*α* as outlined above. Results are shown in Figure 6A, revealing that all are dominantly responsive to HIF-1*α*, and that the minimal (36 bp) promoter appears exclusively responsive to HIF-1*α*.

In further experiments we sought to analyse mechanisms that might underlie the difference between effects of HIF-2*α* on the endogenous PHD3 gene and effects on the juxtaposed enhancer/promoter sequence in PHD3.HRE-PHD3pro-luc. We considered whether altering spacing between the promoter and enhancer might affect HIF-*α* selectivity. The PHD3 enhancer was therefore moved to a more distant position 3′ to the luciferase reporter gene in PHD3pro-luc-PHD3.HRE. Analysis of activity in transiently transfected RCC4 cells again showed that activity was dominantly mediated by HIF-1*α* rather than HIF-2*α* (Figure 6B). We therefore considered the possibility that stable integration into chromatin might be required for selective responses to HIF-2*α*. To address this, we selected stable transfectants of RCC4 cells expressing PHD3pro-luc-PHD3.HRE. However, the activity of the PHD3 enhancer/promoter reporter gene was again suppressed by siRNA directed against HIF-1*α* but not HIF-2*α* (Figure 6C).

## DISCUSSION

Inactivation of the pVHL tumour suppressor is observed in the majority of inherited and sporadic RCC leading to constitutive stabilization of both HIF-1*α* and HIF-2*α* and constitutive upregulation of HIF target gene expression (Kaelin, 2002). However, recent studies have revealed that in this setting, upregulation of HIF-1*α* and HIF-2*α* activates distinct target genes and has contrasting effects on the growth of tumours (Maranchie *et al*, 2002; Kondo *et al*, 2002, 2003; Zimmer *et al*, 2004; Raval *et al*, 2005). These findings imply that strategies for therapeutic blockade of HIF pathways for RCC should most likely attempt to be isoform specific. In this work, we therefore sought to analyse the mechanisms underlying target gene selectivity for HIF-1*α* versus HIF-2*α* in RCC. We performed a detailed analysis of two genes displaying highly contrasting HIF-*α* isoform-specific regulation that is typical of previously analysed patterns of response for two distinct groups of genes. The gene encoding CA9 conforms to a pattern first described among genes encoding glycolytic enzymes (Hu *et al*, 2003), and is specifically responsive to HIF-1*α*, showing no response to HIF-2*α* in any cell type, whether non-RCC or RCC (Grabmaier *et al*, 2004; Raval *et al*, 2005). The gene encoding PHD3 conforms to a pattern previously reported for genes encoding a variety of proteins including VEGF and GLUT1 (Raval *et al*, 2005); it responds dominantly or exclusively to HIF-2*α* in RCC cells, but to both HIF-1*α* and HIF-2*α* in non-RCC cells.

Our findings indicate that this target gene selectivity is achieved by post-DNA-binding mechanisms. Chromatin immunoprecipitation experiments demonstrated non-selective binding for HIF-1*α* versus HIF-2*α* at functional HREs for genes that are selectively responsive to these transcription factors. Furthermore, domain exchange experiments indicated that the ability to activate a target gene was not conferred by exchange of the basic-helix–loop–helix sequences previously implicated in DNA-binding (Jiang *et al*, 1996). Interestingly, despite the ability of transcription factors to bind at sites where they were not transcriptionally active (e.g. HIF-2*α* at the CA9 promoter), siRNA suppression of the inactive isoform did not clearly enhance transcription, suggesting that competition for binding is not important at endogenous levels of the proteins, at least under the circumstances examined.

Further analysis suggested that the post-DNA binding mechanisms activating target gene expression are different for the HIF-1*α* target CA9 and the HIF-2*α* target PHD3. First, the results of the HIF-1*α*/HIF-2*α* domain exchange experiments were non-reciprocal, and implicated non-equivalent sequences in activation of CA9 and PHD3 expression. Second, experiments directed towards defining the *cis*-acting DNA sequences at the CA9 and PHD3 loci that might mediate HIF-α isoform-selective responses yielded quite different results. Thus, for CA9, these experiments suggested that sequences at the minimal promoter could convey selective responses to HIF-1*α*. siRNA-mediated suppression of HIF-*α* isoforms indicated that the minimal CA9 promoter, like the endogenous gene, was specifically responsive to endogenous HIF-1*α* and not HIF-2*α*. Although some breakdown in selectivity was observed under conditions of overexpression by transient transfection, in comparison with other HREs, the CA9 promoter again appeared specifically responsive to HIF-1*α*, suggesting that specificity determining sequences in HIF-1α operate through direct or indirect interactions with factors binding the CA9 promoter.

In contrast, there was no evidence at all of selective activation of the transfected PHD3 HRE by endogenous HIF-2*α*. Rather siRNA suppression experiments revealed that the sequence was dominantly responsive to HIF-1*α*. This was observed irrespective of whether the PHD3 HRE was linked to the PHD3 promoter or a heterologous promoter, for different promoter/HRE spacings, and in both transiently and stably transfected RCC4 cells. Interestingly, in contrast with the lack of effect of siRNA-mediated suppression of HIF-2*α*, overexpressed HIF-2*α* powerfully activated the PHD3 HRE. This unexpected finding has recently been noted by others who have proposed that HIF-2*α* transcriptional activity is downregulated by a titratable repressor that is overcome by overexpression of the molecule (Hu *et al*, 2006). Irrespective of this, our results indicate that despite binding of HIF-2*α* to the native PHD3 HRE in the first intron, there is a marked difference between the activity of HIF-2*α* in promoting transcriptional activity mediated by this sequence, and its activity in promoting expression of the native gene. It is therefore possible that transcriptional activity of HIF-2*α* on the native gene is dependent on ^****^some aspect of this enhancer/promoter configuration, such as spacing that is not accurately reproduced in these experiments, that it is mediated by interaction at other DNA sequences, or that it involves post-transcriptional mechanisms.

Also of interest is the marked shift to dependence on HIF-2*α* in RCC versus non-RCC cells that was observed in these studies for PHD3, and has been previously observed for genes such as VEGF and GLUT1 (Sowter *et al*, 2003; Raval *et al*, 2005). Although this might simply reflect greater abundance of HIF-2*α* versus HIF-1*α* in RCC versus non-RCC cells (Maxwell *et al*, 1999; Krieg *et al*, 2000), or, as illustrated here, the presence of abnormal forms of HIF-1*α* protein in some RCC, there appeared to be a disproportion, for instance between modestly greater HIF-2*α*/HIF-1*α* protein levels in RCC4 versus Hep3B cells, and more striking differences in dependence of PHD3 on HIF-2*α*. This could reflect the existence of mechanisms that restrict the activity of HIF-1*α* or enhance the activity of HIF-2*α* in the RCC context. Though our findings do not distinguish these possibilities, they do indicate that HIF-1*α* is fully active in RCC4 cells in directing transcription from the transfected PHD3 HRE, as in other cell types.

A number of other recent studies have addressed possible mechanisms underlying differential activity of HIF-1*α* versus HIF-2*α* by different approaches. Though an oxidation susceptible cysteine residue in the DNA binding domain of HIF-2*α* has been proposed to mediate differential activity of HIF-2*α* versus HIF-1*α* (Lando *et al*, 2000), it is unlikely that this could account for the post-DNA binding effects we have identified in the current work. More interestingly, the regulatory subunit of I*κ*B kinase (IKK), NF-*κ*B essential modulator (NEMO) has been identified as a HIF-2*α* binding partner that promotes transcriptional activity (Bracken *et al*, 2005). To date however we have not identified any differences in the levels of NEMO in RCC versus non-RCC cells. Whether more subtle changes in NEMO activity exist in RCC cells, and more general analysis of the mode of interaction of HIF-2*α* with its endogenous target genes in RCC will require further investigation. Our studies provide a new focus for such investigations and indicate that they should be directed at post-DNA-binding mechanisms regulating HIF-2*α* target gene expression.
