# Supplementary material for: Target gene selectivity of hypoxia-inducible factor-α in renal cancer cells is conveyed by post-DNA-binding mechanisms
Source: Br J Cancer. 2007 Mar 27;96(8):1284–92. doi: 10.1038/sj.bjc.6603675 (PMC2360163; doi:10.1038/sj.bjc.6603675)
Supplement: Supplementary Table 1 [file 6603675x1.doc]

**Supplementary Table 1**

Primers for mutagenesis

HIF-1α mRNA: NM_001530.2

| **Mutagenesis position** | **Primer orientation** | **Primer sequence** |
| --- | --- | --- |
| c782t (-HindIII)  5’ AAGCTT 3’  aa166 | sense | AAG AAC AAA ACA CAC AGC GAA GTT TTT TTC TCA GAA TGA AGT GTA C |
|  | antisense | GTA CAC TTC ATT CTG AGA AAA AAA CTT CGC TGT GTG TTT TGT TCT T |
| c569t (+HindIII)  5’ AAGCTT 3’  aa95 | sense | ATG AAT TGC TTT TAT TTG AAA GCT TTG GAT GGT TTT GTT ATG GTT CTC |
|  | antisense | GAG AAC CAT AAC AAA ACC ATC CAA AGC TTT CAA ATA AAA GCA ATT CAT |
| a1451g (+HindIII)  5’ AAGCTT 3’  aa389 | sense | AAG TAG CCT CTT TGA CAA GCT TAA GAA GGA ACC TGA TG |
|  | antisense | CAT CAG GTT CCT TCT TAA GCT TGT CAA AGA GGC TAC TT |
| t1515c (+XbaI)  5’ TCTAGA 3’  aa411 | sense | GGA GAC ACA ATC ATA TCT CTA GAT TTT GGC AGC AAC GA |
|  | antisense | TCG TTG CTG CCA AAA TCT AGA GAT ATG ATT GTG TCT CC |
| g2003a, a2006g (+MfeI)  5’ CAATTG 3’  aa574 | sense | CCA ATG GAT GAT GAC TTC CAA TTG CGT TCC TTC GAT CAG TTG |
|  | antisense | CAA CTG ATC GAA GGA ACG CAA TTG GAA GTC ATC ATC CAT TGG |

HIF-2α mRNA: NM_001430.1

| **Mutagenesis position** | **Primer orientation** | **Primer sequence** |
| --- | --- | --- |
| c431t (+HindIII)  5’ AAGCTT 3’  aa94 | sense | ATG GAC AAC TTG TAC CTG AAA GCT TTG GAG GGT TTC ATT GCC |
|  | antisense | GGC AAT GAA ACC CTC CAA AGC TTT CAG GTA CAA GTT GTC CAT |
| C1328t (+HindIII)  5’ AAGCTT 3’  aa393 | sense | TCC TAT TCA CCA AGC TTA AGG AGG AGC CCG AG |
|  | antisense | CTC GGG CTC CTC CTT AAG CTT GGT GAA TAG GA |
| g1391a (+XbaI)  5’ TCTAGA 3’  aa414 | sense | CGC CAT CAT CTC TCT AGA TTT CGG GAA TCA GAA C |
|  | antisense | GTT CTG ATT CCC GAA ATC TAG AGA GAT GAT GGC G |
| g1772a, c1773t, a1775g (+MfeI)  5’ CAATTG 3’  aa542 | sense | CGG GGA AGA CTT CCA ATT GAG CCC CAT CTG CCC C |
|  | antisense | GGG GCA GAT GGG GCT CAA TTG GAA GTC TTC CCC G |

Primers for ChIP analysis

| **Genomic locus** | **Primer orientation** | **Primer sequence** |
| --- | --- | --- |
| PHD3  NT_026437.10 | forward | AGT GTC CGT TCC CAG CTC AG |
|  | reverse | TAG GCA CAG TAA ACA GGC CC |
| CA9  NT_008413.16 | forward | ACC TGC CCC TCA CTC CAC CCC C |
|  | reverse | GCG GCT GAC TGT GGG GTG TCC |
